# The genome sequence of the small nettle, *Urtica urens* L. (Urticaceae)

**DOI:** 10.12688/wellcomeopenres.23187.1

**Published:** 2024-11-01

**Authors:** Maarten J. M. Christenhusz, Alex D. Twyford, Royal Botanic Gardens Kew Genome Acquisition Lab, Plant Genome Sizing collective, Darwin Tree of Life Barcoding collective, Wellcome Sanger Institute Tree of Life Management, Samples and Laboratory team, Wellcome Sanger Institute Scientific Operations: Sequencing Operations, Wellcome Sanger Institute Tree of Life Core Informatics team, Tree of Life Core Informatics collective, Darwin Tree of Life Consortium

**Affiliations:** 1 Curtin University, Perth, Western Australia, Australia; 2 Hortus Botanicus, University of Technology, Delft, The Netherlands; 3 The University of Edinburgh, Edinburgh, Scotland, UK; 4 Royal Botanic Gardens Kew, Richmond, England, UK

**Keywords:** Urtica urens, small nettle, genome sequence, chromosomal, Rosales

## Abstract

We present a genome assembly from a specimen of small nettle, *Urtica urens* (Streptophyta; Magnoliopsida; Rosales; Urticaceae). The genome sequence has a total length of 339.60 megabases. Most of the assembly is scaffolded into 12 chromosomal pseudomolecules. The mitochondrial and plastid genome assemblies have lengths of 335.02 kilobases and 147.51 kilobases, respectively. Gene annotation of this assembly on Ensembl identified 18,378 protein-coding genes.

## Species taxonomy

Eukaryota; Viridiplantae; Streptophyta; Streptophytina; Embryophyta; Tracheophyta; Euphyllophyta; Spermatophyta; Magnoliopsida; Mesangiospermae; eudicotyledons; Gunneridae; Pentapetalae; rosids; fabids; Rosales; Urticaceae; *Urtica*; *Urtica urens* L. (NCBI:txid473050).

## Background

The small nettle, *Urtica urens* L., is a widely distributed plant species that is an ancient introduction to Britain and Ireland (archaeophyte) from mainland Europe. The species is widespread across Eurasia and North and East Africa, but it has become widely naturalised and is now found on all six continents and throughout Britain and Ireland in disturbed habitats ( POWO, 2024). It is common in England, and more scattered in Ireland, Scotland and Wales ( Stroh *et al.*, 2023). It is especially common in urban and agricultural sites, allotments, gardens, waste sites and other frequently disturbed places.

Small nettle is an annual herbaceous plant that is usually small in stature (to 60 cm). It is monoecious with a female biased sex ratio ( Stace *et al.*, 2019). Like many species of *Urtica*, it produces characteristic stinging hairs that are large cells filled with a variety of pain producing compounds such as formic acid ( Thurston & Lersten, 1969), which are assumed to protect against grazing from herbivorous mammals ( Salisbury, 1961). It can cause contact dermatitis (urticaria) in humans, as the large stinging hairs break off and inject the compounds into the skin. Like other nettles, the young leaves are edible as a pot herb after cooking.

The species differs from our native common stinging nettle *U. dioica* in a range of attributes. These include (i) leaf shape and size, as leaves of *U. dioica* are about half the size and more rounded with coarser, deeper toothing than those of *U. dioica*, (ii) life-history and growth form as *U. urens* is a monoecious, herbaceous annual whereas *U. dioica* is a dioecious, stoloniferous perennial, (iii) chromosome number of UK material, as all *U. uren*s individuals counted to date are diploid, with 2 *n* = 2 *x* = 24 ( Henniges *et al.*, 2022; Stace *et al.*, 2019) whereas all *U. dioica* ssp. *dioica* individuals are reported to be tetraploid, typically with 2 *n* = 4 *x* = 52 ( Wentworth *et al.,* 1991), although counts of 2 *n* = 4 *x* = 48 have occasionally been recorded ( Montgomery *et al.,* 1997), and (iv) ecological preferences as *U. urens* prefers drier sites (e.g. sandy arable fields) compared with *U. dioica* ( Boot *et al*., 1986). While *U. urens* is not grazed by most mammals, it is, like most other nettles, an important food plant for many insect species, including caterpillars of the European peacock butterfly, *Aglais io* ( Lohse *et al.*, 2021).

Here, we present the first genome sequence for *Urtica urens*, and for the plant family Urticaceae. It will be useful for further study on the complex taxonomy of the genus *Urtica*.

## Genome sequence report

**Figure 1.**
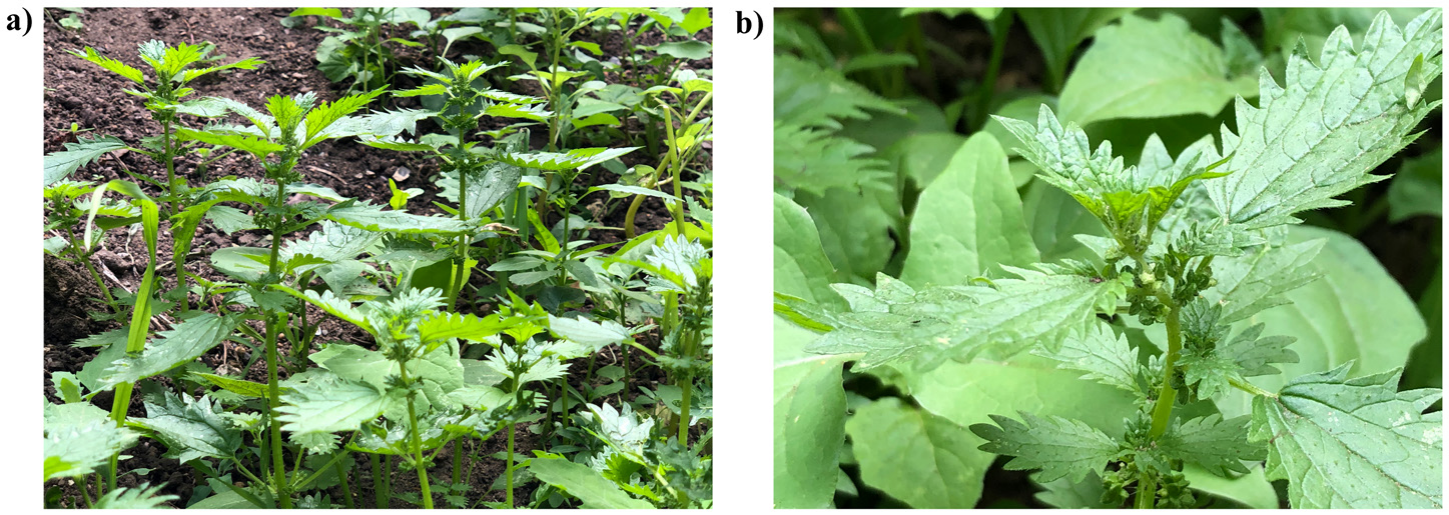
Photographs of the *Urtica urens* (drUrtUren1) specimen used for genome sequencing.

The genome was sequenced from a specimen of *Urtica urens* ( Figure 1) collected from Queen Charlotte’s Cottage, Royal Botanic Gardens, Kew, Richmond, Surrey, UK (51.47, –0.30). Using flow cytometry, the genome size (1C-value) was estimated to be 0.51 pg, equivalent to 500 megabases, although interference from secondary metabolites and the presence of endopolyploidy, made it difficult to be more precise than this. A total of 41-fold coverage in Pacific Biosciences single-molecule HiFi long reads and 122-fold coverage in 10X Genomics read clouds was generated. Primary assembly contigs were scaffolded with chromosome conformation Hi-C data, which produced 123.54 Gb from 818.17 million reads, yielding an approximate coverage of 364-fold. Specimen and sequencing details are provided in Table 1.

**Table 1.** Specimen and sequencing data for *Urtica urens*.

Project information			
**Study title**	*Urtica urens*		
**Umbrella BioProject**	PRJEB47318		
**BioSample**	SAMEA7521823		
**NCBI taxonomy ID**	473050		
Specimen information			
**Technology**	**ToLID**	**BioSample accession**	**Organism part**
**PacBio long read sequencing**	drUrtUren1	SAMEA7521889	leaf
**Hi-C sequencing**	drUrtUren1	SAMEA7521888	leaf
**RNA sequencing**	drUrtUren1	SAMEA7521889	leaf
Sequencing information			
**Platform**	**Run accession**	**Read count**	**Base count (Gb)**
**Hi-C Illumina NovaSeq 6000**	ERR6688734	8.18e+08	123.54
**PacBio Sequel II**	ERR6939265	2.22e+06	18.86
**Chromium Illumina NovaSeq 6000**	ERR6688731	1.55e+08	23.41
**Chromium Illumina NovaSeq 6000**	ERR6688732	1.38e+08	20.9
**Chromium Illumina NovaSeq 6000**	ERR6688733	1.17e+08	17.72
**Chromium Illumina NovaSeq 6000**	ERR6688730	1.22e+08	18.43
**RNA Illumina NovaSeq X**	ERR13148232	5.21e+07	7.87
**RNA Illumina NovaSeq X**	ERR13148233	5.10e+07	7.7
**RNA Illumina NovaSeq X**	ERR12861024	5.82e+07	8.79
**RNA Illumina NovaSeq X**	ERR13148231	5.58e+07	8.43
**RNA Illumina NovaSeq X**	ERR13148234	5.25e+07	7.93
**RNA Illumina HiSeq 4000**	ERR6688735	4.78e+07	7.22

**Figure 2.**
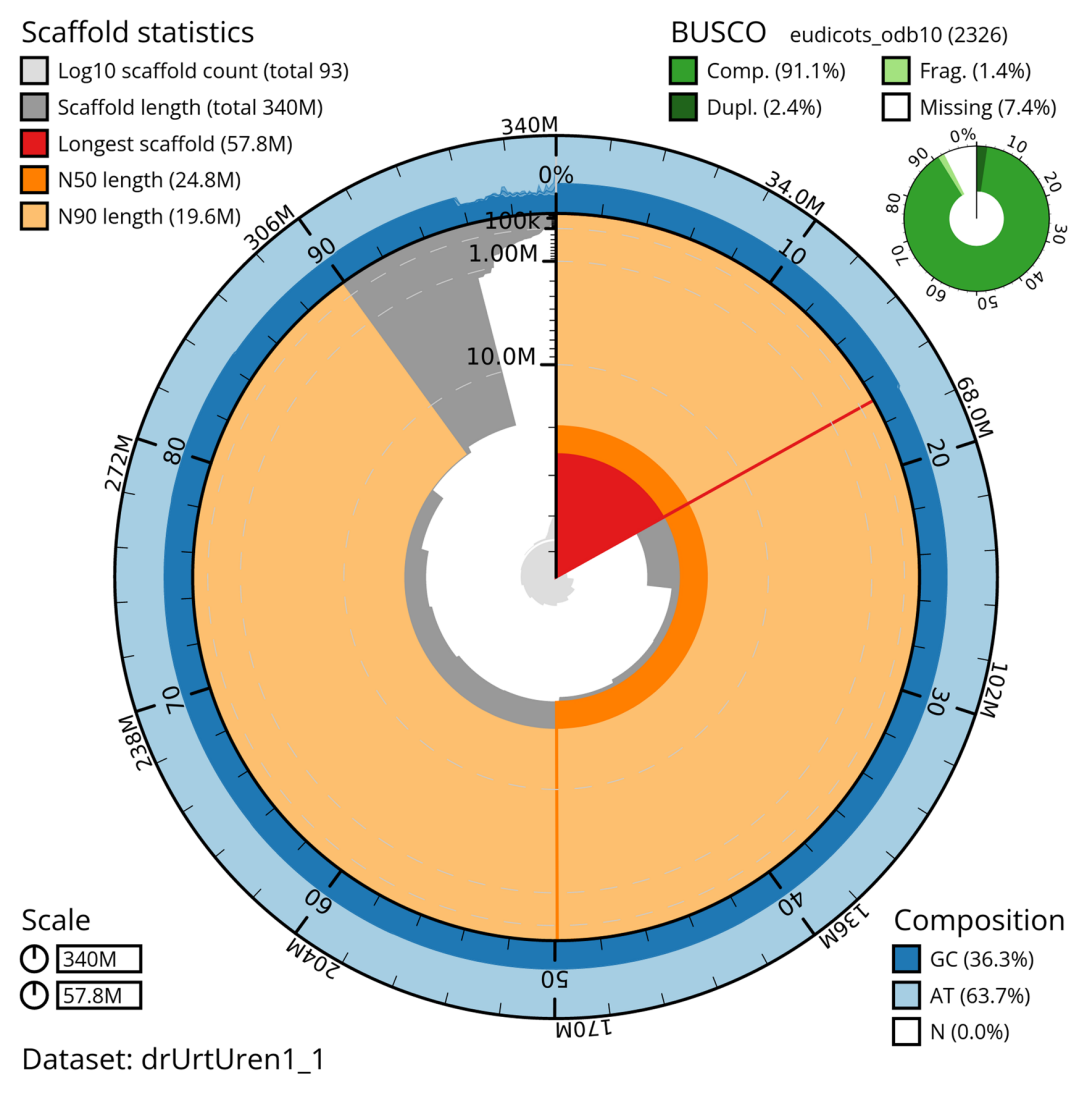
Genome assembly of *Urtica urens*, drUrtUren1.1: metrics. The BlobToolKit snail plot shows N50 metrics and BUSCO gene completeness. The main plot is divided into 1,000 size-ordered bins around the circumference with each bin representing 0.1% of the 340,129,727 bp assembly. The distribution of scaffold lengths is shown in dark grey with the plot radius scaled to the longest scaffold present in the assembly (57,787,223 bp, shown in red). Orange and pale-orange arcs show the N50 and N90 scaffold lengths (24,801,648 and 19,612,646 bp), respectively. The pale grey spiral shows the cumulative scaffold count on a log scale with white scale lines showing successive orders of magnitude. The blue and pale-blue area around the outside of the plot shows the distribution of GC, AT and N percentages in the same bins as the inner plot. A summary of complete, fragmented, duplicated and missing BUSCO genes in the eudicots_odb10 set is shown in the top right. An interactive version of this figure is available at https://blobtoolkit.genomehubs.org/view/Urtica%20urens/dataset/drUrtUren1_1/snail.

**Figure 3.**
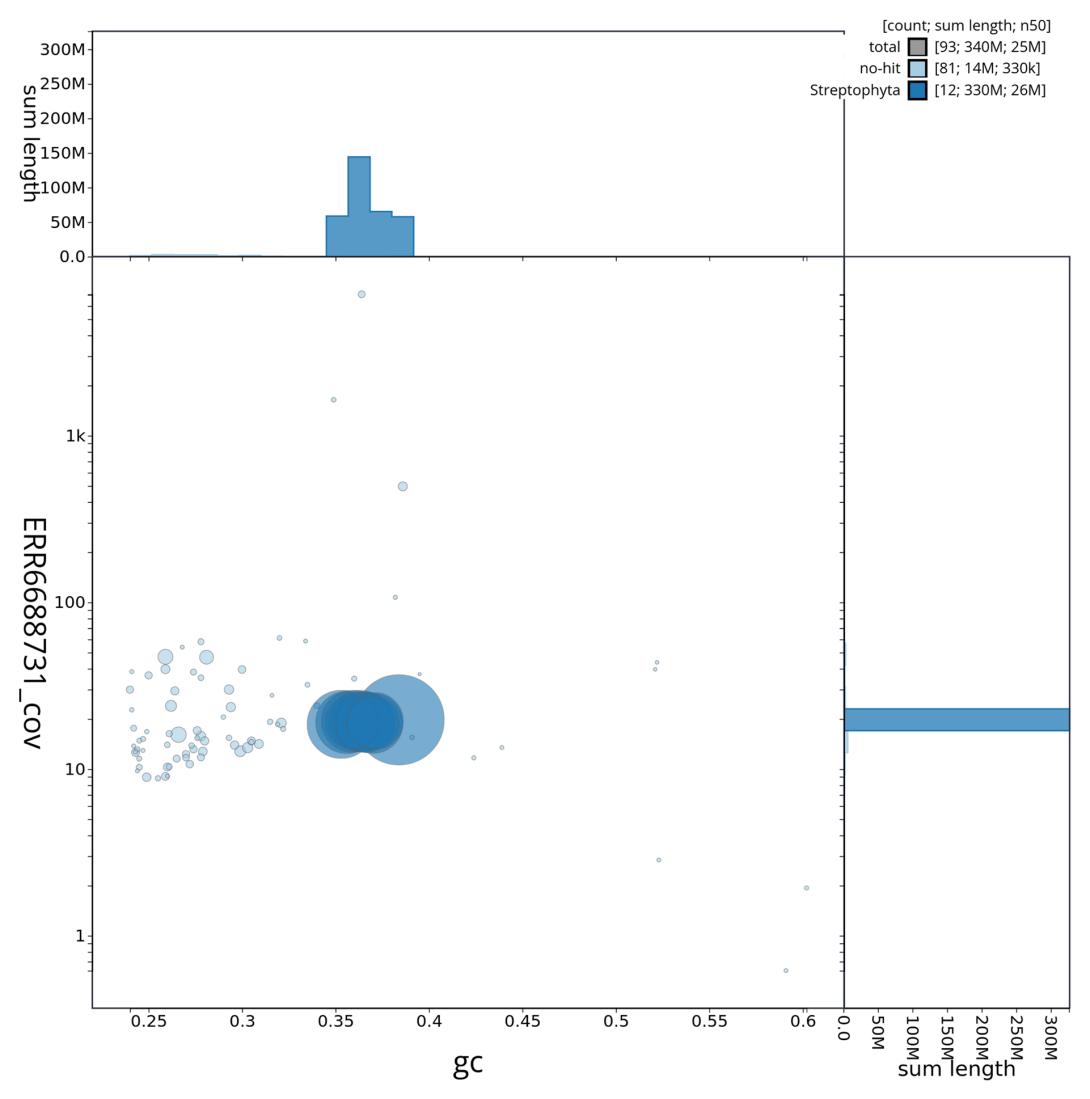
Genome assembly of *Urtica urens*, drUrtUren1.1: BlobToolKit GC-coverage plot. Scaffolds are coloured by phylum. Circles are sized in proportion to scaffold length. Histograms show the distribution of scaffold length sum along each axis. An interactive version of this figure is available at https://blobtoolkit.genomehubs.org/view/Urtica%20urens/dataset/drUrtUren1_1/blob.

**Figure 4.**
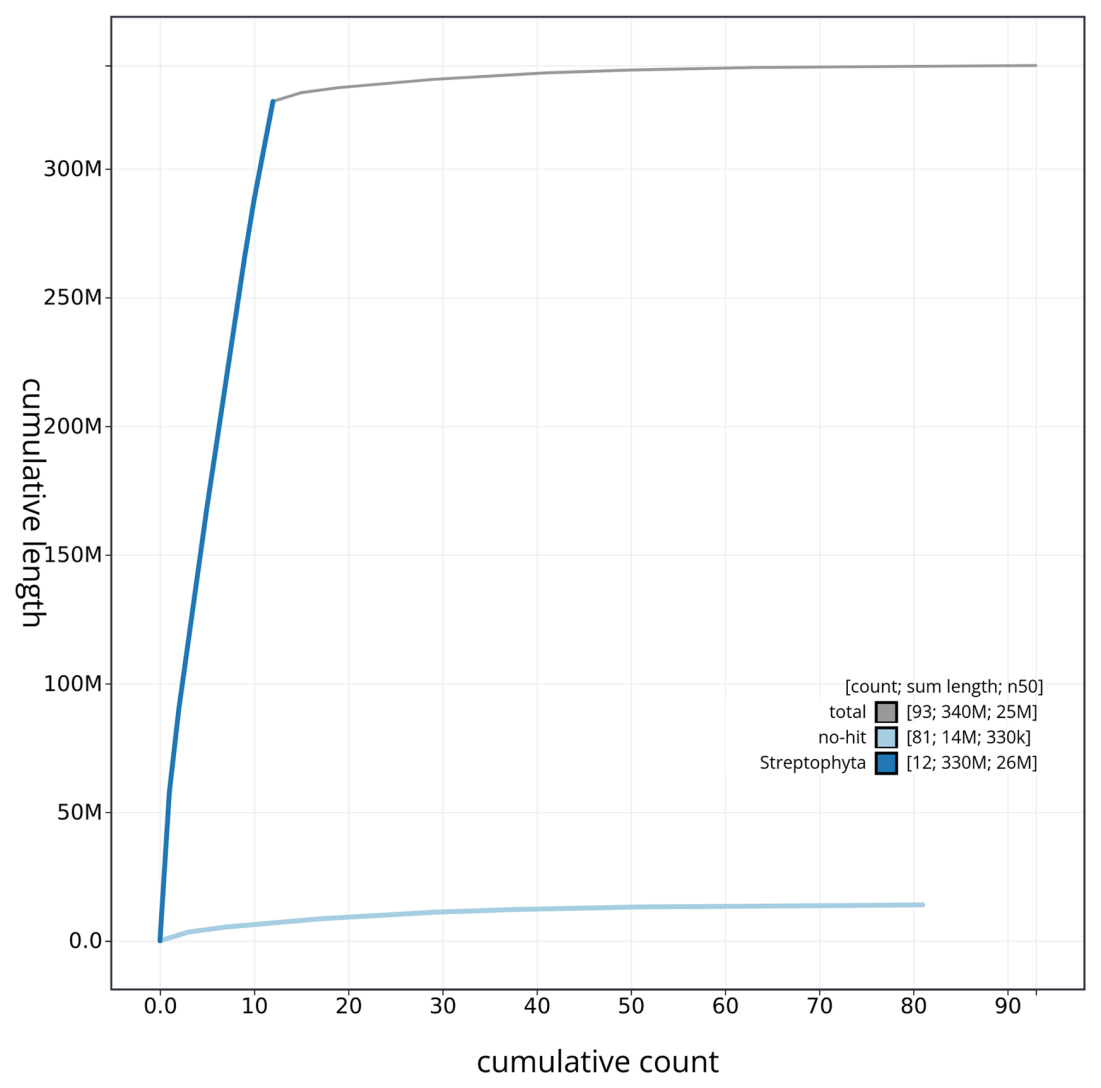
Genome assembly of *Urtica urens*, drUrtUren1.1: BlobToolKit cumulative sequence plot. The grey line shows cumulative length for all scaffolds. Coloured lines show cumulative lengths of scaffolds assigned to each phylum using the buscogenes taxrule. An interactive version of this figure is available at https://blobtoolkit.genomehubs.org/view/Urtica%20urens/dataset/drUrtUren1_1/cumulative.

**Figure 5.**
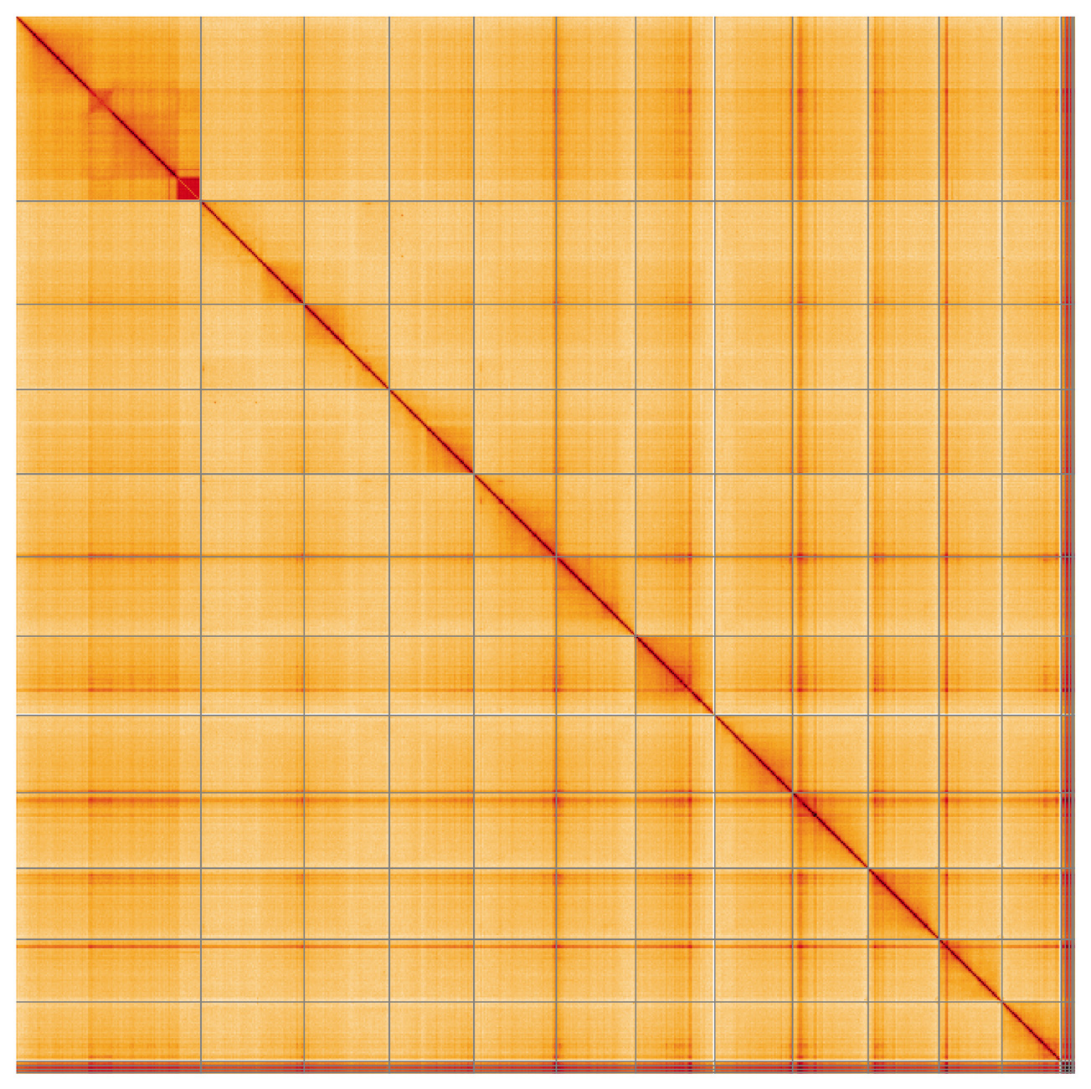
Genome assembly of *Urtica urens*, drUrtUren1.1: Hi-C contact map of the drUrtUren1.1 assembly, visualised using HiGlass. Chromosomes are shown in order of size from left to right and top to bottom. An interactive version of this figure may be viewed at https://genome-note-higlass.tol.sanger.ac.uk/l/?d=RrD1vKb1Rdy6v8Asj6wMaA.

Manual assembly curation corrected 244 missing joins or mis-joins and removed one haplotypic duplication, reducing the scaffold number by 73.04%, and increasing the scaffold N50 by 2.77%. The final assembly has a total length of 339.60 Mb in 91 sequence scaffolds with a scaffold N50 of 24.8 Mb ( Table 2) with 269 gaps. The snail plot in Figure 2 provides a summary of the assembly statistics, while the distribution of assembly scaffolds on GC proportion and coverage is shown in Figure 3. The cumulative assembly plot in Figure 4 shows curves for subsets of scaffolds assigned to different phyla. Most (95.89%) of the assembly sequence was assigned to 12 chromosomal-level scaffolds. Chromosome-scale scaffolds confirmed by the Hi-C data are named in order of size ( Figure 5; Table 3). Chromosome 1 has a highly repetitive region of low confidence from ~50.4 Mb–57.7 Mb where the order and orientation of the scaffolds cannot be confirmed. While not fully phased, the assembly deposited is of one haplotype. Contigs corresponding to the second haplotype have also been deposited. The mitochondrial and plastid genomes were also assembled and can be found as contigs within the multifasta file of the genome submission.

**Table 2.** Genome assembly data for *Urtica urens*, drUrtUren1.1.

Genome assembly		
Assembly name	drUrtUren1.1	
Assembly accession	GCA_958296335.1	
*Accession of alternate haplotype*	*GCA_958296315.1*	
Span (Mb)	339.60	
Number of contigs	362	
Contig N50 length (Mb)	19.9	
Number of scaffolds	91	
Scaffold N50 length (Mb)	24.8	
Longest scaffold (Mb)	57.79	
Assembly metrics *		*Benchmark*
Consensus quality (QV)	54.1	*≥ 50*
*k*-mer completeness	99.98%	*≥ 95%*
BUSCO **	C:91.1%[S:88.8%,D:2.4%], F:1.4%,M:7.4%,n:2,326	*C ≥ 95%*
Percentage of assembly mapped to chromosomes	95.89%	*≥ 95%*
Organelles	Mitochondrial genome: 335.02 kb; plastid genome: 147.51 kb	*complete single alleles*
Genome annotation at Ensembl		
Number of protein-coding genes	18,378	
Number of non-coding genes	6,146	
Number of gene transcripts	30,750	

* Assembly metric benchmarks are adapted from column VGP-2020 of “Table 1: Proposed standards and metrics for defining genome assembly quality” from Rhie *et al.* (2021). ** BUSCO scores based on the eudicots_odb10 BUSCO set using version 5.4.3. C = complete [S = single copy, D = duplicated], F = fragmented, M = missing, n = number of orthologues in comparison. A full set of BUSCO scores is available at https://blobtoolkit.genomehubs.org/view/drUrtUren1_1/dataset/drUrtUren1_1/busco.

**Table 3.** Chromosomal pseudomolecules in the genome assembly of *Urtica urens*, drUrtUren1.

INSDC accession	Name	Length (Mb)	GC%
OY282520.1	1	57.79	38.5
OY282521.1	2	32.17	35.5
OY282522.1	3	26.56	35.5
OY282523.1	4	26.44	36.0
OY282524.1	5	25.71	36.5
OY282525.1	6	24.8	36.5
OY282526.1	7	24.7	37.0
OY282527.1	8	24.13	36.5
OY282528.1	9	23.63	36.5
OY282529.1	10	22.14	37.0
OY282530.1	11	19.61	36.0
OY282531.1	12	18.51	37.0
OY282532.1	MT	0.34	38.5
OY282533.1	Pltd	0.15	36.5

The estimated Quality Value (QV) of the final assembly is 54.1 with *k*-mer completeness of 99.98%, and the assembly has a BUSCO v5.4.3 completeness of 91.1% (single = 88.8%, duplicated = 2.4%), using the eudicots_odb10 reference set ( *n* = 2,326).

Metadata for specimens, BOLD barcode results, spectra estimates, sequencing runs, contaminants and pre-curation assembly statistics are given at https://links.tol.sanger.ac.uk/species/473050.

## Genome annotation report

The *Urtica urens* genome assembly (GCA_958296335.1) was annotated at the European Bioinformatics Institute (EBI) on Ensembl Rapid Release. The resulting annotation includes 30,750 transcribed mRNAs from 18,378 protein-coding and 6,146 non-coding genes ( Table 2; https://rapid.ensembl.org/Urtica_urens_GCA_958296335.1/Info/Index). The average transcript length is 2,480.03. There are 1.25 coding transcripts per gene and 4.91 exons per transcript.

## Methods

### Sample acquisition, DNA barcoding and genome size estimation

An individual *Urtica urens* (specimen ID KDTOL10014, ToLID drUrtUren1) was picked by hand from near Queen Charlotte’s Cottage, Royal Botanic Gardens, Kew, Richmond, Surrey, UK (latitude 51.47, longitude –0.30) on 2020-08-05. The specimen was collected and identified by Maarten J. M. Christenhusz (Royal Botanic Gardens, Kew) and preserved by freezing at –80 °C. The herbarium voucher associated with the sequenced plant (K001400687) was deposited in the herbarium of the Royal Botanic Gardens, Kew (K).

The initial species identification was verified by an additional DNA barcoding process according to the framework developed by Twyford *et al*. (2024). Part of the plant specimen was preserved in silica gel desiccant. A DNA extraction from the dried plant was amplified by PCR for standard barcode markers, with the amplicons sequenced and compared to public sequence databases including GenBank and the Barcode of Life Database (BOLD). The barcode sequences for this specimen are available on BOLD ( Ratnasingham & Hebert, 2007). Following whole genome sequence generation, DNA barcodes were also used alongside the initial barcoding data for sample tracking through the genome production pipeline at the Wellcome Sanger Institute ( Twyford *et al.*, 2024). The standard operating procedures for the Darwin Tree of Life barcoding have been deposited on protocols.io ( Beasley *et al.*, 2023).

The genome size was estimated by flow cytometry using the fluorochrome propidium iodide and following the ‘one-step’ method as outlined in Pellicer *et al.* (2021). For this species, the General Purpose Buffer (GPB) supplemented with 3% PVP and 0.08% (v/v) beta-mercaptoethanol was used for isolation of nuclei ( Loureiro *et al.*, 2007), and the internal calibration standard was *Solanum lycopersicum* ‘Stupiké polní rané’ with an assumed 1C-value of 968 Mb ( Doležel *et al.*, 2007).

### Nucleic acid extraction

The workflow for high molecular weight (HMW) DNA extraction at the Wellcome Sanger Institute (WSI) Tree of Life Core Laboratory includes a sequence of core procedures: sample preparation and homogenisation, DNA extraction, fragmentation and purification. Detailed protocols are available on protocols.io ( Denton *et al.*, 2023).

The drUrtUren1 sample (leaf tissue) was weighed and dissected on dry ice ( Jay *et al.*, 2023), and was then cryogenically disrupted using the Covaris cryoPREP ^®^ Automated Dry Pulverizer ( Narváez-Gómez *et al.*, 2023). HMW DNA was extracted using the Automated Plant MagAttract v1 protocol ( Sheerin *et al.*, 2023). HMW DNA was sheared into an average fragment size of 12–20 kb in a Megaruptor 3 system ( Todorovic *et al.*, 2023). Sheared DNA was purified by solid-phase reversible immobilisation, using AMPure PB beads to eliminate shorter fragments and concentrate the DNA ( Strickland *et al.*, 2023). The concentration of the sheared and purified DNA was assessed using a Nanodrop spectrophotometer and Qubit Fluorometer and Qubit dsDNA High Sensitivity Assay kit. Fragment size distribution was evaluated by running the sample on the FemtoPulse system.

RNA was extracted from leaf tissue of drUrtUren1 in the Tree of Life Laboratory at the WSI using the RNA Extraction: Automated MagMax™ *mir*Vana protocol ( do Amaral *et al.*, 2023). The RNA concentration was assessed using a Nanodrop spectrophotometer and a Qubit Fluorometer using the Qubit RNA Broad-Range Assay kit. Analysis of the integrity of the RNA was done using the Agilent RNA 6000 Pico Kit and Eukaryotic Total RNA assay.

### Library preparation and sequencing

Pacific Biosciences HiFi circular consensus and 10X Genomics read cloud DNA sequencing libraries were constructed according to the manufacturers’ instructions. Poly(A) RNA-Seq libraries were constructed using the NEB Ultra II RNA Library Prep kit. DNA and RNA sequencing was performed by the Scientific Operations core at the WSI on Pacific Biosciences Sequel II (HiFi) and Illumina NovaSeq 6000 (10X) and Illumina HiSeq 4000 (RNA-Seq) instruments.

Hi-C data were generated from the leaf tissue of drUrtUren11 using the Arima-HiC v2 kit. Tissue was finely ground using cryoPrep and then subjected to nuclei isolation using the Qiagen QProteome Kit. After isolation, the nuclei were fixed, and the DNA crosslinked using pure formaldehyde. The crosslinked DNA was then digested using a restriction enzyme master mix. The 5’-overhangs were filled in and labelled with a biotinylated nucleotide, followed by proximity ligation. The biotinylated DNA constructs were fragmented to a size of 400 to 600 bp using a Covaris E220 sonicator. The DNA was then enriched, barcoded, and amplified using the NEBNext Ultra II DNA Library Prep Kit, following the manufacturer's instructions. Hi-C sequencing was performed using paired-end sequencing with a read length of 150 bp on an Illumina NovaSeq 6000 instrument.

### Genome assembly, curation and evaluation

***Assembly***

The HiFi reads were first assembled using Hifiasm ( Cheng *et al.*, 2021) with the --primary option. Haplotypic duplications were identified and removed with purge_dups ( Guan *et al.*, 2020). One round of polishing was performed by aligning 10X Genomics read data to the assembly with Long Ranger ALIGN, calling variants with FreeBayes ( Garrison & Marth, 2012). Hi-C reads were further mapped with bwa-mem2 ( Vasimuddin *et al.*, 2019) to the primary contigs. The assembly was then scaffolded with Hi-C data ( Rao *et al.*, 2014) using SALSA2 ( Ghurye *et al.*, 2019). Scaffolded assemblies were evaluated using Gfastats ( Formenti *et al.*, 2022), BUSCO ( Manni *et al.*, 2021) and MERQURY.FK ( Rhie *et al.*, 2020). The organelle genomes were assembled using OATK ( Zhou, 2023).

***Curation***

The assembly was checked for contamination and corrected using the gEVAL system ( Chow *et al.*, 2016). Manual curation was primarily conducted using PretextView ( Harry, 2022), with additional insights provided by JBrowse2 ( Diesh *et al.*, 2023) and HiGlass ( Kerpedjiev *et al.*, 2018). Scaffolds were visually inspected and corrected as described by Howe *et al*. (2021). Any identified contamination, missed joins, and mis-joins were corrected, and duplicate sequences were tagged and removed. The process is documented at https://gitlab.com/wtsi-grit/rapid-curation (article in preparation).

***Evaluation of final assembly***

To assess the assembly metrics, the *k*-mer completeness and QV consensus quality values were calculated in Merqury ( Rhie *et al.*, 2020). This work was done using the “sanger-tol/readmapping” ( Surana *et al.*, 2023a) and “sanger-tol/genomenote” ( Surana *et al.*, 2023b) pipelines. The genome readmapping pipelines pipelines were developed using nf-core tooling ( Ewels *et al.*, 2020) and MultiQC ( Ewels *et al.*, 2016), relying on the Conda package manager, the Bioconda initiative ( Grüning *et al.*, 2018), the Biocontainers infrastructure ( da Veiga Leprevost *et al.*, 2017), as well as the Docker ( Merkel, 2014) and Singularity ( Kurtzer *et al.*, 2017) containerisation solutions. The genome was also analysed within the BlobToolKit environment ( Challis *et al.*, 2020) and BUSCO scores ( Manni *et al.*, 2021) were calculated.

Table 4 contains a list of relevant software tool versions and sources.

**Table 4.** Software tools: versions and sources.

Software tool	Version	Source
BlobToolKit	4.2.1	https://github.com/blobtoolkit/blobtoolkit
BUSCO	5.3.2	https://gitlab.com/ezlab/busco
bwa-mem2	2.2.1	https://github.com/bwa-mem2/bwa-mem2
Cooler	0.8.11	https://github.com/open2c/cooler
FreeBayes	1.3.1-17- gaa2ace8	https://github.com/freebayes/freebayes
Gfastats	1.3.6	https://github.com/vgl-hub/gfastats
Hifiasm	0.15.3	https://github.com/chhylp123/hifiasm
HiGlass	1.11.6	https://github.com/higlass/higlass
Long Ranger ALIGN	2.2.2	https://support.10xgenomics.com/genome-exome/ software/pipelines/latest/advanced/other-pipelines
Merqury	MerquryFK	https://github.com/thegenemyers/MERQURY.FK
OATK	0.2	https://github.com/c-zhou/oatk
PretextView	0.2	https://github.com/wtsi-hpag/PretextView
purge_dups	1.2.3	https://github.com/dfguan/purge_dups
SALSA	2.2	https://github.com/salsa-rs/salsa
sanger-tol/ genomenote	v1.0	https://github.com/sanger-tol/genomenote
sanger-tol/ readmapping	1.1.0	https://github.com/sanger-tol/readmapping/tree/1.1.0

### Wellcome Sanger Institute – Legal and Governance

The materials that have contributed to this genome note have been supplied by a Darwin Tree of Life Partner. The submission of materials by a Darwin Tree of Life Partner is subject to the **‘Darwin Tree of Life Project Sampling Code of Practice’**, which can be found in full on the Darwin Tree of Life website here. By agreeing with and signing up to the Sampling Code of Practice, the Darwin Tree of Life Partner agrees they will meet the legal and ethical requirements and standards set out within this document in respect of all samples acquired for, and supplied to, the Darwin Tree of Life Project.

Further, the Wellcome Sanger Institute employs a process whereby due diligence is carried out proportionate to the nature of the materials themselves, and the circumstances under which they have been/are to be collected and provided for use. The purpose of this is to address and mitigate any potential legal and/or ethical implications of receipt and use of the materials as part of the research project, and to ensure that in doing so we align with best practice wherever possible. The overarching areas of consideration are:

•   Ethical review of provenance and sourcing of the material

•   Legality of collection, transfer and use (national and international)

Each transfer of samples is further undertaken according to a Research Collaboration Agreement or Material Transfer Agreement entered into by the Darwin Tree of Life Partner, Genome Research Limited (operating as the Wellcome Sanger Institute), and in some circumstances other Darwin Tree of Life collaborators.

## Data Availability

European Nucleotide Archive: *Urtica uren*s. Accession number PRJEB47318; https://identifiers.org/ena.embl/PRJEB47318 ( Wellcome Sanger Institute, 2023). The genome sequence is released openly for reuse. The *Urtica urens* genome sequencing initiative is part of the Darwin Tree of Life (DToL) project. All raw sequence data and the assembly have been deposited in INSDC databases. Raw data and assembly accession identifiers are reported in Table 1.

## Author information

Members of the Royal Botanic Gardens Kew Genome Acquisition Lab are listed here: https://doi.org/10.5281/zenodo.12625079.

Members of the Plant Genome Sizing collective are listed here: https://doi.org/10.5281/zenodo.7994306.

Members of the Darwin Tree of Life Barcoding collective are listed here: https://doi.org/10.5281/zenodo.12158331

Members of the Wellcome Sanger Institute Tree of Life Management, Samples and Laboratory team are listed here: https://doi.org/10.5281/zenodo.12162482.

Members of Wellcome Sanger Institute Scientific Operations: Sequencing Operations are listed here: https://doi.org/10.5281/zenodo.12165051.

Members of the Wellcome Sanger Institute Tree of Life Core Informatics team are listed here: https://doi.org/10.5281/zenodo.12160324.

Members of the Tree of Life Core Informatics collective are listed here: https://doi.org/10.5281/zenodo.12205391.

Members of the Darwin Tree of Life Consortium are listed here: https://doi.org/10.5281/zenodo.4783558.
