# Supplementary material for: The genome sequence of the small nettle, Urtica urens L. (Urticaceae)
Source: Wellcome Open Res. 2024 Nov 1;9:639. [Version 1] doi: 10.12688/wellcomeopenres.23187.1 (PMC11612553; doi:10.12688/wellcomeopenres.23187.1)
Supplement: Peer review history

# Reviewer report: Reviewer response for version 1

**DOI:** 10.21956/wellcomeopenres.25537.r109377

**Published:** 2024-12-02

**Authors:** Oluwaseyi Shorinola

**Affiliations:** 1 University of Birmingham, Birmingham, UK

The manuscript by Christenhusz et al. describe the genome assembly and annotation of *Urtrica urens* - a nettle plant that is widely distributed in the United Kingdom. The authors combine Pacbio sequencing with 10x genomic sequencing to make a base assembly which was then scaffolded using chromosome conformation. Before the sequencing, the authors used flow cytometry to estimate the genome size of *U. urens*. Lastly, the authors used the Ensembl rapid release pipeline to identify coding and non-coding gene models in the *U uren* assembly.

 The rationale for creating the dataset is clear. As the first genome assembly from the *Utrica* genus, the resource will be useful for genetic and genomic analyses of the genus. Also, the protocol and method used are appropriate.

 However, there are some missing information and issues that the author must address:

- Using flow cytometry, the authors estimate the genome size of the *U. urens* to be ~500 Mb but the genome assembly only span 339.6 Mb which is about 68% of the estimated genome size. The authors should discuss possible reasons for this difference. Could the 500 Mb be an over-estimation due to the interference experienced during genome size estimation. A suggestion would be to also attempt kmer-based genome size estimation.
- Secondly, the author should explain or clarify how the read coverage values were estimated. It seems that the HiC coverage value (364x) is based on dividing the total HiC base count (123.54 Gb) by the genome assembly length (339.60 Mbp). But this is not ideal, the coverage should be based on estimated genome size. Also, it is not clear how coverage was derived for the PacBio and 10x reads.
- The authors describe RNA extractions and sequencing experiments but did not describe how this data was used. I presume that it was used as input for Ensembl gene annotation. The authors should clarify this.
- In addition to the BUSCO analysis for the genome, the authors should conduct similar BUSCO analysis to assess the quality of the gene models.  Although the quality of the genome assembly and gene annotation are often correlated, the quality of the later also depends on the robustness of the gene annotation pipeline used.  
- The authors should properly describe the a and b panels of Figure 1.
- The number of scaffolds is stated as 91 in Table 2 but 93 in Figure 2.  Also, in the legend of Figure 2, the assembly length is stated 340,129,727 bp but as 339.6 Mb in Table 2. The authors should clarify these discrepancies.
- In Figure 2, there are two scales on the bottom left corner. It is not clear how these scales are different or which part of the figure the scales apply to.

Are sufficient details of methods and materials provided to allow replication by others?

Partly

Is the rationale for creating the dataset(s) clearly described?

Yes

Are the datasets clearly presented in a useable and accessible format?

Yes

Are the protocols appropriate and is the work technically sound?

Yes

Reviewer Expertise:

Plant genetics and genomics.

I confirm that I have read this submission and believe that I have an appropriate level of expertise to confirm that it is of an acceptable scientific standard, however I have significant reservations, as outlined above.

# Reviewer report: Reviewer response for version 1

**DOI:** 10.21956/wellcomeopenres.25537.r110636

**Published:** 2024-11-25

**Authors:** Richard Edwards

**Affiliations:** 1 University of Western Australia, Perth, Australia

This is a well-described Genome Note with the core methods and assembly features described. The introduction frames the work nicely and provides good background on the study species. The genome itself appears to be of high quality. Overall, the manuscript is well written, with just a few minor points of clarification needed here and there.

 REVISIONS/SUGGESTIONS

1. Abstract: “Most of the assembly is scaffolded into 12 chromosomal pseudomolecules.” It would be better to provide the actual figure, and highlight that the karyotype is 2 *n* = 24 (i.e. correct).
2. Abstract: Were the mitochondrial and plastid genomes complete and (if expected) circular?
3. The genome size needs a bit more discussion and clarity. The flow cytometry estimate is nearly 50% bigger than the assembly. In my experience, the assembly is probably more accurate, but it would be good to confirm this. A kmer-based and/or depth-based genome size prediction should be reported to verify this? Are the reported coverage stats for sequencing based on 500 Mbp or 340 Mbp (or something else, like empirical read depth)? Are there any signs of massive regions of collapsed repeats? The 500 Mbp size was based on a standard "with an assumed 1C-value of 968 Mb”. What are the error margins of this assumed size? Is this sufficient to account for the discrepancy?
4. I am bit confused as the manuscript first describes the assembly as a scaffolded primary assembly, and then implies that it is one haplotype. Hifiasm will produce three assemblies in “primary” mode: a primary, and TWO phased haplotypes. The primary usually contains part of both haplotypes, and thus achieves higher completeness than either haplotype alone. It is complemented by “alternative” contigs. Please confirm whether it was indeed the primary assembly used and, if so, how the phasing was checked with respect to the “second haplotype” deposited. Please provide diploid Merqury plots that show whether there are parts of the “second haplotype” falsely duplicated (in the context of a phased assembly) in the primary haplotype.
5. A k-mer completeness of 99.98% is extremely high. Is this the value for both haplotypes combined? If not, is heterozygosity really low? (Is this individual highly in-bred?)
6. Figure 5. What’s at the end of chr1? Is it where the rRNA repeats are?
7. It would be nice to have telomere predictions to get a sense of how many chromosomes are full-length. Is anything known about the number of chromosome arms in the karyotype? (Telocentric and acrocentric chromosomes often will not assemble telomeres at the ends.)

Are sufficient details of methods and materials provided to allow replication by others?

Yes

Is the rationale for creating the dataset(s) clearly described?

Yes

Are the datasets clearly presented in a useable and accessible format?

Yes

Are the protocols appropriate and is the work technically sound?

Yes

Reviewer Expertise:

Biodiversity genomics, genome assembly, genome curation, bioinformatics.

I confirm that I have read this submission and believe that I have an appropriate level of expertise to confirm that it is of an acceptable scientific standard, however I have significant reservations, as outlined above.

# Reviewer report: Reviewer response for version 1

**DOI:** 10.21956/wellcomeopenres.25537.r110627

**Published:** 2024-11-20

**Authors:** Saneyoshi Ueno

**Affiliations:** 1 Forestry and Forest Products Research Institute, Tsukuba, Japan

This article presents the genome assembly of the small nettle, *Urtica urens*, marking the first genome sequencing for this species. The sequencing involved multiple methods: PacBio HiFi, 10X Genomics read clouds, and Hi-C data, resulting in a high-coverage assembly. The article provides detailed methodology, and the genome sequence data and assembled genome are available in public repositories.

 My specific comments are as follows:

 Q1: Could you provide details on the QC of raw sequencing reads, specifically whether quality/adapter trimming was performed or not, and if so, how it was conducted?

 Q2: For the options used in analysis programs, if no specific values are mentioned, does this mean the default settings were used? If so, it might be beneficial to state this explicitly.

 Q3: Where are the scaffold (contig) sequences other than the 12 chromosomes, chloroplast, and mitochondrial genomes uploaded? It would be preferable if these scaffolds were also made available.

 Q4: The following sentence contains "pipelines" twice, so one instance should be deleted:

 "The genome readmapping pipelines pipelines were developed using nf-core tooling....."

Are sufficient details of methods and materials provided to allow replication by others?

Yes

Is the rationale for creating the dataset(s) clearly described?

Yes

Are the datasets clearly presented in a useable and accessible format?

Yes

Are the protocols appropriate and is the work technically sound?

Yes

Reviewer Expertise:

Forest Genetics

I confirm that I have read this submission and believe that I have an appropriate level of expertise to confirm that it is of an acceptable scientific standard.
